# Digit-Induced Globe Rupture in a Patient With Mixed Personality Disorder: A Rare Form of Self-Harm

**DOI:** 10.1192/bjo.2026.11786

**Published:** 2026-06-30

**Authors:** Avgoustinos Ioannou, Olivia Baggaley, Islam Ahmed, Sowmya Maiya

**Affiliations:** 1 Derbyshire Healthcare NHS Foundation Trust, Derbyshire, United Kingdom; 2 University Hospitals of Derby and Burton NHS Foundation Trust, Derby, United Kingdom

## Abstract

**Aims:**

This case presentation explores an extreme form of self-harm by M, a woman in her late fifties, with historical diagnosis of schizophrenia, later revised to mixed personality disorder with anxious-avoidant, impulsive and antisocial traits. She experienced significant trauma in childhood in the form of physical abuse by father, needing hospital admission. Additionally, she has multiple medical comorbidities including Multiple Sclerosis and multiple transient ischaemic attacks, and two previous incidents of self-harm, including an intentional overdose requiring intensive care and tracheostomy. She has received trazodone and flupentixol depot intermittently since the 1980s, stopped in November 2025 by a covering consultant, against M’s wishes, due to absence of psychotic symptoms and worsening of physical health comorbidities.

**Methods:**

M was admitted to the ward informally after presenting to the Emergency Department with suicidal ideation and thoughts of jumping in front of a lorry if not admitted. She quickly became comfortable in the ward setting but was noted to have personality changes compared to the community, including selective engagement with staff members. She became dependent on staff for Activities of Daily Living (ADLs), despite Occupational Therapy deeming her able of independent ADLs. Her behaviour became disruptive and challenging, including stripping naked, spreading faeces, and pulling her hair. This escalated to M attempting to remove her left eye from the orbit with her fingers, requiring an emergency lateral canthotomy and emergency repair of her ruptured globe in theatre, and resulted in loss of vision from that eye. M appeared to enjoy the attention she received during this incident. She has since attempted to remove her right eye, requiring close observation and frequent interventions by staff to prevent this.

**Results:**

M’s complex, lengthy psychiatric presentation and physical health comorbidities resulted in challenges in her management. Her presentation appears to be on the neurotic rather than psychotic spectrum, with antipsychotics prescribed for agitation. She has periods of lucidity before dissociation, with bizarre, disinhibited behaviour. Her distressed family queried the possibility of Munchausen Syndrome. Early severe trauma can present with challenging behavioural traits, emotional dysregulation and fight-flight response, leading to fluctuating withdrawal and aggression depending on context and perceived unmet needs.

**Conclusion:**

This case is an example of a complex patient with multiple diagnostic and management challenges which culminated in rare and extreme self-harm resulting in loss of sight. It highlights the importance of collateral history and multi-disciplinary approach regarding diagnosis and management, contributing to the literature on mixed personality disorder.

